# The Associations of Single Nucleotide Polymorphisms in miR-146a, miR-196a and miR-499 with Breast Cancer Susceptibility

**DOI:** 10.1371/journal.pone.0070656

**Published:** 2013-09-09

**Authors:** Ping-Yu Wang, Zong-Hua Gao, Zhong-Hua Jiang, Xin-Xin Li, Bao-Fa Jiang, Shu-Yang Xie

**Affiliations:** 1 Department of Epidemiology, Binzhou Medical University, YanTai, ShanDong, P.R. China; 2 Department of Epidemiology, School of Public Health, Shandong University, JiNan, ShanDong, P.R. China; 3 Department of Imaging, Yantai Traditional Chinese Medical Science Hospital, YanTai, ShanDong, P.R. China; Sanjay Gandhi Medical Institute, India

## Abstract

**Background:**

Previous studies have investigated the association between single nucleotide polymorphisms (SNPs) located in microRNAs (miRNAs) and breast cancer susceptibility; however, because of their limited statistical power, many discrepancies are revealed in these studies. The meta-analysis presented here aimed to identify and characterize the roles of miRNA SNPs in breast cancer risk, and evaluate the associations of polymorphisms in miR-146a rs2910164, miR-196a rs11614913 and miR-499 rs3746444 with breast cancer susceptibility, respectively.

**Methodology/Principal Findings:**

The PubMed and Embases databases were searched updated to 31^st^ December, 2012. The complete data of polymorphisms in miR-146a rs2910164, miR-196a rs11614913 and miR-499 rs3746444 from case-control studies for breast cancer were analyzed by odds ratios (ORs) with 95% confidence intervals (CIs) to reveal the associations of SNPs in miRNAs with breast cancer susceptibility. Totally, six studies for rs2910164 in miR-146a, involving 4225 cases and 4469 controls; eight studies for rs11614913 in miR-196a, involving 4110 cases and 5100 controls; and three studies of rs3746444 in miR-499, involving 2588 cases and 3260 controls, were investigated in the meta-analysis. The rs11614913 (TT+CT) genotype of miR-196a2 was revealed to be associated with a decreased breast cancer susceptibility compared with the CC genotypes (OR = 0.906, 95% CI: 0.825–0.995, *P* = 0.039); however, no significant associations were observed between rs2910164 in miR-146a (or rs3746444 in miR-499) and breast cancer susceptibility.

**Conclusions:**

This meta-analysis demonstrates the compelling evidence that the rs11614913 CC genotype in miR-196a2 increases breast cancer risk, which provides useful information for the early diagnosis and prevention of breast cancer.

## Introduction

MicroRNAs (miRNAs) are non-coding RNA molecules that can act as tumor suppressor genes or oncogenes [1]. There are more than 1000 miRNA genes in the human genome [2]–[4], which regulate the translation or degradation of human messenger RNA (mRNA) by sequence complementarity [5]–[7]. MiRNAs regulate approximately 30% of human genes [8]. The genetic variants of a miRNA may affect its biogenesis and maturation [9], [10], which are causally linked to the pathogenesis of numerous diseases, including cancer [11], [12].

Several miRNA polymorphisms have been reported to affect miRNA processing or miRNA-mRNA interactions [12], [13]. Single nucleotide polymorphisms (SNPs) in miRNAs can be used as genetic markers to predict breast cancer susceptibility or prognosis. For example, a significant association was identified between polymorphism rs11614913 in miR-196a2 and breast cancer risk [14]. Breast cancer patients with the variant C allele in miR-146a produced higher levels of mature miR-146, which may predispose women to an earlier age of onset of familial breast cancer [15], [16]. The variant genotypes rs3746444 in miR-499 were also reported to be associated with significantly increased risks of breast cancer [17]. The rs6505162 with the CC genotype in miR-423 could reduce the risk of breast cancer development [18]. Nevertheless, some SNPs in miRNAs showed no association with breast cancer risk [19], [20]. Catucci *et al.* reported that the SNPs rs11614913 in miR-196a2, rs3746444 in mir-499 and rs2910164 in miR-146a were not related to breast cancer risk [19]. Jedlinski's study also did not support the association of polymorphism rs11614913 in miR-196a2 with breast cancer susceptibility [20]. Thus, there are many discrepancies concerning the relationship between SNPs in miRNA (miR-146a, miR-196a2, and miR-499) and breast cancer susceptibility, which may be attributed to sample sizes, different ethnic group and different miRNAs studied.

Meta-analysis is statistical methods for contrasting and combining results from different studies, in the hope of identifying sources of disagreement among those results [21]. A meta-analysis allows derivation and statistical testing of overall factors and effect-size parameters, which can identify whether a publication bias exists or whether the results are more varied than what is expected from the sample diversity. Though several meta-analysis studies evaluating the roles of miRNA gene polymorphisms in cancer have been published, few meta-analysis studies have assessed the associations of three SNPs of miR-146a, miR-196a and miR-499 with breast cancer susceptibility. Therefore, we selected these three SNPs in this meta-analysis, according to two basic principles as established in a previous study [22]: first, the minor allele frequency of the SNP was not less than 5%; Secondly, only functional SNPs were selected. This meta-analysis aimed to resolve the discrepancies among the results of the associations of these miRNAs (miR-146a, miR-196a2, and miR-499) with breast cancer susceptibility.

## Materials and Methods

### Eligible studies and data extraction

PubMed and Embases databases were searched with the following terms: “breast cancer/carcinoma”, “polymorphism/variant”, “miR-146a/rs2910164”, “miR-196a2/rs11614913” or “miR-499/rs3746444”. The searched articles, published in English language updated to 31^st^ December, 2012, were limited to human species, female sex and cancer subjects of adult patients (19+ years). All the titles and abstracts of searched articles were reviewed to exclude clearly irrelevant studies. The full texts of the remaining articles were read, and a manual search of the references from original studies was performed to identify additional articles of the same topic.

All the case-control studies were studied according to the Strengthening the Reporting of Observational Studies in Epidemiology (STROBE) as a previous report [23]. The specific inclusion criteria as follows: (1) Case-control studies: cases are patients newly diagnosed with breast cancer, and the controls were subjects without breast cancer; (2) Odds ratios (ORs) with their 95% confidence intervals (95% CIs) are calculated from correct and sufficient polymorphism distribution data; (3) Correct statistical analysis. The strict exclusion criteria were: (1) Pure cell studies, non-breast cancer studies; (2) Articles that are not case-control studies; (3) Repeated or overlapped studies; (4) Articles with obvious mistakes.

Two reviewers (Zong-Hua Gao and Xin-Xin Li) extracted data independently using standardized forms. The following characteristics were collected from each study if available: (1) publication year; (2) first author's name; (3) country origin; (4) ethnicity were categorized as Caucasian, and non-Caucasian; (5) genotyping methods; (6) total numbers of cases and controls; (7) miR-146a, miR-196a and miR-499 polymorphism distribution data, respectively; (8) *P* value for Hardy-Weinberg equilibrium (HWE) of controls. In case of disagreement, another reviewer (Zhong-Hua Jiang) resolved these disagreements according to the original data.

### Statistical analysis

Deviation in the controls of all studies from HWE tests were carried out online using a web-based program (http://ihg.gsf.de/cgi-bin/hw/hwa1.pl), and *P* value <0.05 was considered significant. The ORs with their corresponding 95% CIs (homozygote comparison, heterozygote comparison, dominant model and recessive model, respectively) were calculated to analyze the associations of polymorphisms (rs2910164 in miR-146, rs11614913 in miR-196a2 and rs3746444 in miR-499) with breast cancer susceptibility. The significance of the pooled ORs was checked by the *Z* test, and statistical significance was defined as *P* value <0.05.

Cochran *Q* test and estimating *I^2^* test were used to evaluate whether the results from these studies were homogeneous [24], [25]. For Cochran *Q* test, *P* value <0.10 suggests heterogeneity among studies. As *I^2^* test, *I^2^* value <40% indicates “not important heterogeneity”, while a value >75% shows “considerable heterogeneity”. If presence of heterogeneity, the random effects model (DerSimonian Laird) was chosen. Otherwise, the fixed effects model (Mantel-Haenszel) was appropriately used to calculate the pooled ORs.

Publication bias was evaluated using the Begg-Mazumdar adjusted rank correlation test and the Egger regression asymmetry test, *P* value <0.10 was considered as the representative of statistically significant publication bias [26], [27]. Sensitivity analysis was carried to assess the stability of these results. All Statistical analyses were carried out using STATA 11.0 software (STATA Corp, College Station, TX, USA).

## Results

### Characteristics of studies

**Figure 1:**
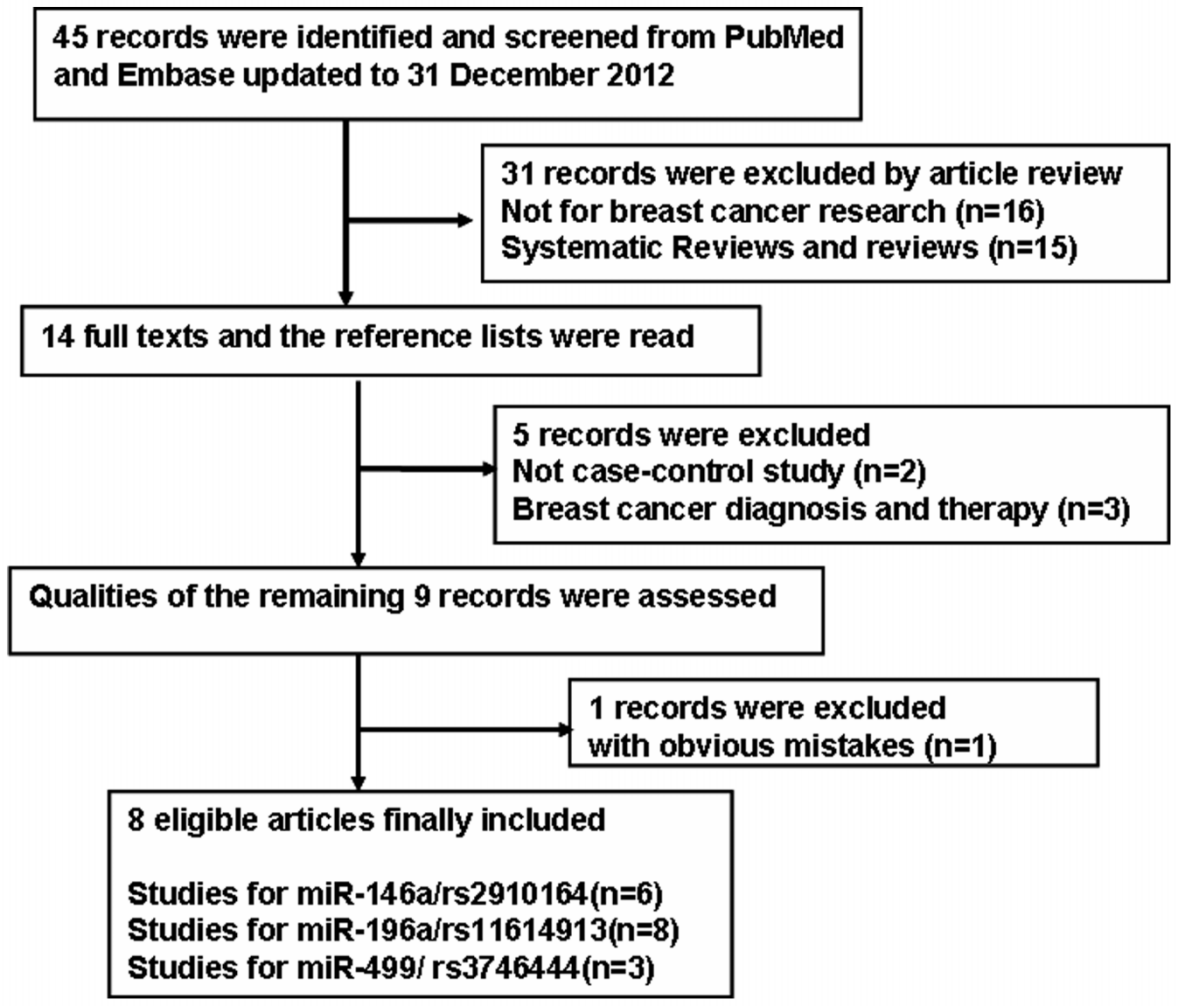
Flow diagram of selection of studies with criteria for inclusion and exclusion.

Eligible studies were selected according to the inclusion and exclusion criteria (Figure 1). Thirty-one records were excluded by reviewing article titles and abstracts, including 16 records that did not focus on breast cancer and 15 records that were systematic reviews. Then, 14 full texts and related reference lists were read. Five records were excluded: 2 records were not case-control studies and 3 records were breast cancer diagnosis and therapy studies. The article published by Alshatwi contained discrepancies between the data shown in the tables and the data described in the results section [14]; therefore, after consultation with the author, these data were excluded. In Catucci's [19] and Linhares's [28] studies, the genotype frequencies were presented according to the subjects' country or race, as in previous reports [29], [30]; thus in the present analysis each group was considered as an independent study. Moreover, in some included articles, if two or more miRNA SNPs were investigated in an article, each miRNA SNP was considered as an independent study. Therefore, six studies, involving 4225 cases and 4469 controls, were ultimately analyzed for the SNP (rs2910164) in miR-146a [15], [17], [19], [31], [32]; eight studies, involving 4110 cases and 5100 controls, were performed for rs11614913 in miR-196a [17], [19], [20], [22], [28], [32]; and three studies, involving 2588 cases and 3260 controls, were tested for rs3746444 in miR-499 [17], [19], respectively.

Characteristics of the included studies were shown in Table 1. These studies were published from year 2009 to 2012. The subjects came from different countries (Australia, Brazil, China, France, Germany, Italy, and USA). Ethnicity was categorized as Caucasians or non-Caucasians population. Genotyping methods included polymerase chain reaction-restriction fragment length polymorphism (PCR-RFLP), TaqMan SNP genotyping assay and MassArray multiplex. Blood samples were used for genotyping in most studies. HWE was assessed by a chi-square test. The distribution of genotypes in the controls agreed with HWE (*P*>0.05) in most of the studies, but some parts of the data in Catucci's [19] and Linhares's [28] studies significantly departed from HWE (*P*<0.05). Begg-Mazumdar adjusted rank correlation test and the Egger regression asymmetry test were used to assess the publication bias of the currently available literature.

**Table 1:** Characteristics of the studies in the meta-analysis.

Year	Study	Country	Ethnicity	Genotyping method	Case	Control	Case			Control			P_HWE_
miR-146a rs2910164							GG	CG	CC	GG	CG	CC	
2011	Garcia et al	France	Caucasian	Taqman	1130	596	676	388	66	352	220	24	0.150
2010	Catucci et al	Germany	Caucasian	Taqman	805	904	451	304	50	536	318	50	0.753
2010	Catucci et al	Italy	Caucasian	Taqman	754	1243	409	286	59	650	520	73	0.019
2010	Pastrello et al	Italy	Caucasian	Taqman	88	155	53	30	5	90	59	6	0.332
2009	Hoffman et al	USA	Caucasian	MassARRAY	439	478	234	176	29	273	178	27	0.775
2009	Hu et al	China	non-Caucasian	PCR-RFLP	1009	1093	165	515	329	180	551	362	0.221

miR-196a2 rs11614913							CC	CT	TT	CC	CT	TT	
2012	Zhang et al	China	non-Caucasian	PCR-RFLP	248	243	11	89	148	17	93	133	0.893
2012	Linhares et al	Brazil	non-Caucasian	PCR-RFLP	63	114	11	29	23	33	51	30	0.264
2012	Linhares et al	Brazil	Caucasian	PCR-RFLP	325	274	83	148	94	94	114	66	0.008
2011	Jedlinski et al	Australia	Caucasian	PCR-RFLP	187	171	68	86	33	58	82	31	0.830
2010	Catucci et al	Germany	Caucasian	Taqman	1101	1496	432	512	157	584	696	216	0.711
2010	Catucci et al	Italy	Caucasian	Taqman	751	1243	334	330	87	532	550	161	0.315
2009	Hoffman et al	USA	Caucasian	MassARRAY	426	466	181	209	36	166	229	71	0.583
2009	Hu et al	China	non-Caucasian	PCR-RFLP	1009	1093	239	483	287	218	517	358	0.210

mir-499 rs3746444							GG	AG	AA	GG	AG	AA	
2010	Catucci et al	Germany	Caucasian	Taqman	823	925	37	250	536	34	290	601	0.893
2010	Catucci et al	Italy	Caucasian	Taqman	756	1242	47	295	414	86	452	704	0.250
2009	Hu et al	China	Asian	PCR-RFLP	1009	1093	44	258	707	29	248	816	0.057

### Main results

The meta-analysis results of the three SNPs in the miRNAs and breast cancer risk were shown in Table 2. There were no significant associations between polymorphisms rs2910164 in miR-146a and breast cancer susceptibility for all genetic models. Because the data for the Italy population group in Catucci's study [19] significantly departed from HWE (*P* = 0.019), we deleted the data to analyze the associations of rs2910164 in miR-146a with breast cancer susceptibility; no significant risk associations were observed between them.

**Table 2:** Meta-analysis results for the four polymorphisms and breast cancer risk. (OR, odds ratio; CI, confidence interval.)

Has-mir-146a (rs2910164)	No. of studies	Sample size (cases/controls)	?2	P_-H_	*I^2^*(%)	Model	OR(95%CI)	z	P_-z_
C vs G	6	4225/4469	2.29	0.808	0.0	F	1.036(0.968–1.108)	1.02	0.308
CC vs GG	6	4225/4469	2.63	0.757	0.0	F	1.156(0.980–1.364)	1.72	0.085
CC vs CG	6	4225/4469	6.26	0.282	20.1	F	1.103(0.955–1.274)	1.33	0.183
CG+CC vs GG	6	4225/4469	3.92	0.562	0.0	F	1.022(0.932–1.120)	0.46	0.644
CC vs GG+CG	6	4225/4469	4.80	0.441	0.0	F	1.102(0.960–1.264)	1.38	0.168
has-mir-196a (rs11614913)									
T vs C	8	4110/5100	25.36	0.001	72.5	R	0.994(0.875,1.129)	0.10	0.924
TT vs CC	8	4110/5100	25.65	0.001	72.7	R	0.970(0.738–1.275)	0.22	0.828
CT vs CC	8	4110/5100	9.97	0.190	29.8	F	0.970(0.882–1.067)	0.63	0.530
TT vs CT+CC	8	4110/5100	17.68	0.013	60.4	R	0.952(0.791–1.147)	0.51	0.609
TT+CT vs CC	8	4110/5100	17.83	0.013	60.7	R	0.987(0.836–1.165)	0.15	0.877
has-mir-499 (rs3746444)									
G vs A	3	2588/3260	4.34	0.114	53.9	F	1.100(0.960–1.260)	1.37	0.171
GG vs AA	3	2588/3260	4.17	0.124	52.0	F	1.194(0.931–1.532)	1.40	0.162
AG vs AA	3	2588/3260	2.24	0.327	10.6	F	1.090(0.972–1.223)	1.48	0.139
GG vs AA+AG	3	2588/3260	4.31	0.116	53.5	F	1.156(0.905–1.477)	1.16	0.247
GG+AG vs AA	3	2588/3260	2.95	0.229	32.1	F	1.107(0.992–1.235)	1.81	0.070

**Figure 2:**
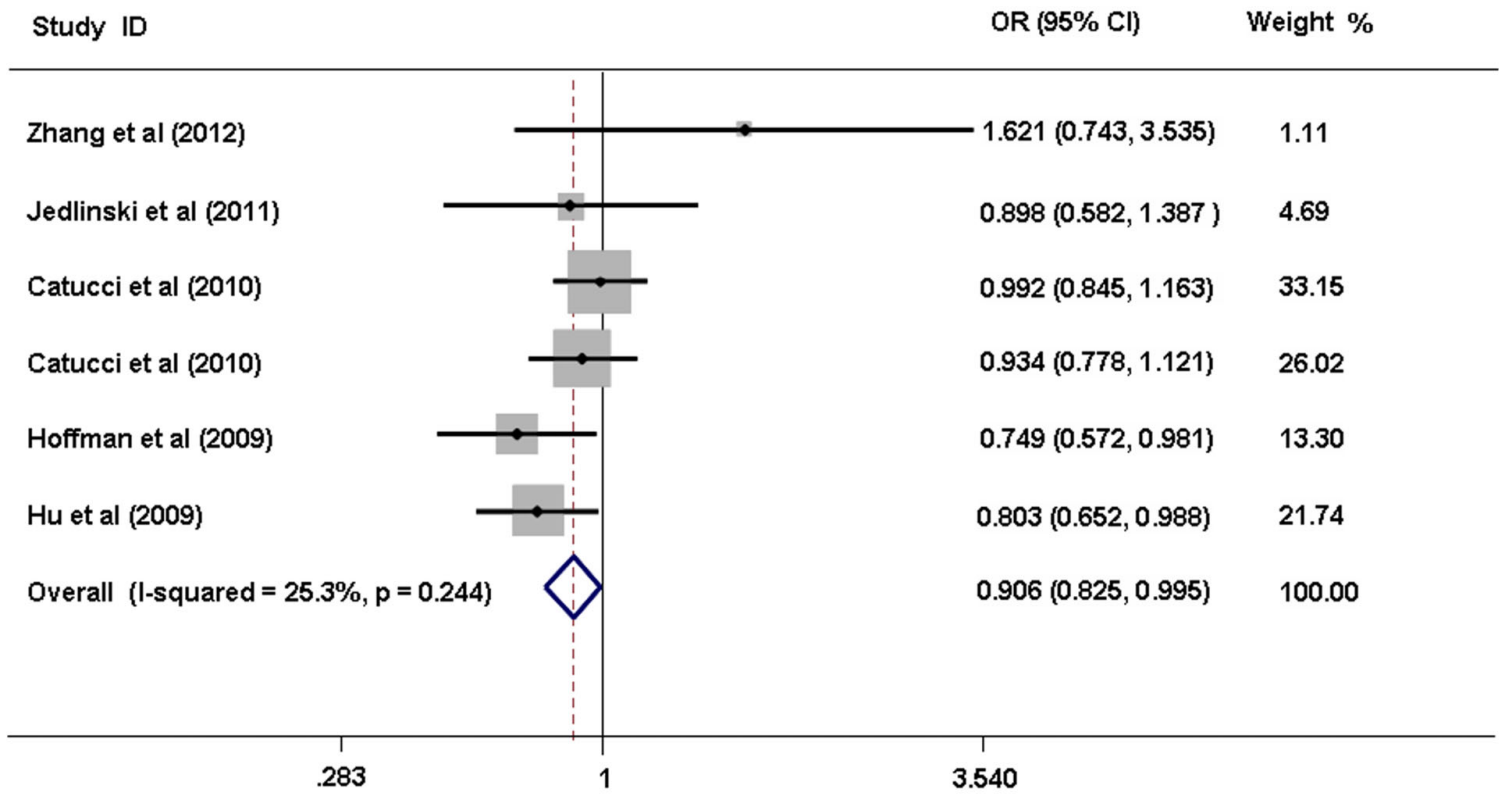
Forest plot for the association between miR-196a2 polymorphism and breast cancer risk. (Significant difference was observed for the comparison of miR-196a2 polymorphism (TT+CT) vs. CC using a fixed-effects model. OR, odds ratio; CI, confidence interval.)

When all the studies concerning SNP rs11614913 in miR-196a2 were pooled into this meta-analysis, no significant breast cancer risk was observed for any SNP genotype of miR-196a2. After excluding the Linhares's study [28], in which the distribution of miR-196a2 genotypes in controls deviated from the HWE (*P* = 0.008) and the included population was mixed, we found that the heterogeneities of the miR-196a2 SNP data were reduced and the genotypic results were more credible. In the comparision of genotypes (TT+CT) *vs* CC, obvious heterogeneity (Heterogeneity chi-square test = 17.83, *P-_Het_* = 0.013, *I^2^* = 60.7%) was reduced to little heterogeneity (Heterogeneity chi-square test = 6.70, *P*-*_Het_* = 0.244, *I^2^* = 25.3%). Then, the fixed effect model was used and a significant difference was observed between the (TT+CT) genotype and breast cancer susceptibility (OR 0.906, 95% CI: 0.825–0.995, *P* = 0.039, Figure 2). No significant risk associations with breast cancer susceptibility were demonstrated for the other SNP genotypes.

Three studies of polymorphism rs3746444 in miR-499 were included in the meta-analysis. No significant risk associations with breast cancer susceptibility were revealed for any SNP of the miR-499 genotypes. No subgroup analysis was performed for the limited studies.

Significant heterogeneities in the data of miR-196a2 rs11614913 SNPs were observed in Table 2. Then sources of this heterogeneity were evaluated systematically using meta-regression. The source of heterogeneity was found to be mainly related to the article publication year (t = 4.64, *P* = 0.004). Because the limit of the published article number, we did not perform the subgroup analysis by publication year.

All the results for the three SNPs in the miRNAs obstained from random model or fixed model were similar. No publication bias was found in this meta-analysis using Begg's (*P*>0.05) and Egger's tests (*P*>0.05).

## Discussion

MiRNAs have been linked to the etiology, progression and prognosis of cancer [33]. The gain or loss of SNPs in miRNA genes often affect the targeting gene function through the transcription [12]. To date, many studies have investigated the roles of SNPs in miRNAs in breast cancer susceptibility [14]–[18], [22], [31], [34], [35]. Among them, SNPs rs2910164 in miR-146a [36]–[38], rs11614913 in miR-196-a2 [14], [17], [29], [39], [40] and rs3746444 in miR-499 [14], [19], [41] are three SNPs that are commonly found in mature miRNA regions, which may contribute to breast cancer susceptibility. However, the results remain contradictory and inconclusive [14], [19], [20], [31]. Therefore, in this meta-analysis, we further explored the associations of these three SNPs (in miR-146a, miR-196-a2 and miR-499) with breast cancer risk.

Polymorphism rs2910164 in miR-146a is located in the 3p strand and comprises a G to C change, which results in a change from a G∶U pair to a C∶U mismatch in the stem structure of the miR-146a precursor and alters the expression of mature miR-146a to influence cancer risk [42], [43]. To further explore whether miR-146a rs2910164 is associated with breast cancer susceptibility, 4225 cases and 4469 controls are investigated for miR-146a rs2910164 in this meta-analysis. Our results failed to find an association between polymorphism rs2910164 in miR-146a and breast cancer risk, similar to other studies [37], [38], [44], but is different to Lian's report, which showed that increased risk of breast cancer was associated with the CC genotype of rs2910164 in miR-146a in Europeans [36]. The difference between our study and Lian's study may be attributed to removing or taking the Italy population data in Catucci's study [19]. In our study, we found that the Italy population data in Catucci's study deviated from the HWE (*P* = 0.019) and removed this data from our meta-analysis. But, this data was calculated in Europeans in Lian's study [36].

Polymorphism rs11614913 in miR-196a2, which is located in the 3′ mature sequence of miR-196a2, may affect pre-miRNA maturation [12], [17]. Li *et al.* reported that the expression level of miR-196a was significantly higher in hepatocellular carcinoma patients with the CC genotype (or at least one C genotype) than in patients with the TT genotype [45]. Many studies showed that individuals carrying the CC genotype could suffer from significantly elevated the risk of breast cancer, lung cancer, gastric cancer, colorectal cancer and hepatocellular carcinoma compared to those with TT or TT+TC genotypes [44]–[46]. When all eligible studies were pooled into this meta-analysis, no significantly increased breast cancer risk was found. After excluding the data in which genotype distribution in the controls deviated from the HWE, the heterogeneities were reduced, revealing an association of the CC genotype of miR-196a2 SNP with an increased breast cancer risk compared with the TT+CT genotypes, which was consistent with our previous finding [47]. Our results provide the compelling evidence that polymorphism rs11614913 in miR-196a2 plays a crucial role in breast cancer development, and supports the view this SNP in miR-196a2 could be used as a candidate biomarker for the diagnosis of breast cancer risk.

Polymorphism rs3746444 in miR-499 involves an A to G nucleotide substitution, which leads to a change from an A∶U pair to a G∶U mismatch in the stem structure of the miR-499 precursor [48]. A number of case-control studies have investigated the association of SNP in miR-499 with cancer risk in multiple types of cancer [46], [48], [49]. However, only a few epidemiological studies focused on the association between polymorphisms rs3746444 in miR-499 and breast cancer risk. Our meta-analysis failed to discover an obvious association between rs3746444 in miR-499 and breast cancer risk. The exact roles of miR-499 SNPs in breast cancer risk require further studies.

Sample size is an important parameter for investigating the genetic effect of any SNP. Our meta-analysis provided higher and sufficient numbers of cases and controls than a single study, significantly increasing the statistical power. In addition, we assessed the qualities of the studies in this meta-analysis, which improved the reliability of the results.

Although meta-analysis is robust, there are still several limitations in this study. First, our study did not evaluate any potential gene-gene interaction and gene-environment interactions. Second, our analysis was based on English publications, which may have introduced a language bias. Last, a lack of sufficient eligible studies limited further subgroup analyses.

In conclusion, this study demonstrates that SNP rs11614913 in miR-196a2 plays a crucial role in the development of breast cancer. We found no significant associations of polymorphisms rs2910164 in miR-146 and rs3746444 in miR499 with breast cancer susceptibility. Well-designed studies with larger sample sizes are needed to confirm the roles of these miRNA polymorphisms in breast cancer risk.

## Supporting Information

Checklist S1 **PRISMA 2009 Checklist for this Meta-analysis.**
(DOC)
